# PAH contamination in Beijing’s topsoil: A unique indicator of the megacity’s evolving energy consumption and overall environmental quality

**DOI:** 10.1038/srep33245

**Published:** 2016-09-16

**Authors:** Jinguo Li, Yi Zheng, Xiaolin Luo, Zhongrong Lin, Wei Zhang, Xuejun Wang

**Affiliations:** 1 Department of Energy and Resources Engineering, College of Engineering, Peking University, Beijing 100871, China; 2 School of Environmental Science and Engineering, South University of Science and Technology, Shenzhen 518055, Guangdong Province, China; 3 School of Environment and Natural Resources, Renmin University of China, Beijing 100872, China; 4 MOE Laboratory of Earth Surface Processes, College of Urban and Environmental Sciences, Peking University, Beijing 100871, China

## Abstract

To improve its air quality, Beijing, the capital of China, has implemented high-cost pollution control measures mainly focused on shifting its energy mix. However, the effectiveness of these measures has long been questioned, especially given the recent problem of severe haze. The main study objectives are to achieve independent, although indirect, information on Beijing’s air pollution by measuring the level of polycyclic aromatic hydrocarbon (PAH) contamination in topsoil and to examine how soil contamination reflects energy consumption. Soil sampling data from two years, 2004 and 2013, were used. The key findings are as follows: 1) although the total PAH content in the topsoil did not significantly decrease from 2004 to 2013, the composition changed considerably; 2) as of 2013, vehicle emissions replaced coal combustion as the leading source of soil PAHs, which validates the existing policy measures regarding vehicle purchasing and traffic volume; 3) the regional transport of atmospheric pollutants, as indicated by the contribution of coking sources in 2013, is not negligible; and 4) appropriate policy measures are needed to control the growing practice of burning biomass. Overall, this study demonstrates that the PAH contamination in topsoil represents an informative indicator of Beijing’s energy consumption and overall environmental quality.

Rapid economic and urban growth in developing countries has degraded air quality and perhaps increased human health risks^1^,^2^,^3^ along a trajectory similar to that previously experienced by developed nations^4^. Recently, severe haze events in China’s megacities have drawn global attention^5^ and prompted a national campaign against PM_2.5_ pollution^6^. In Beijing, haze has been observed more often and with increasing severity^7^,^8^. In the winter of 2015–2016, Beijing suffered severe and frequent multiday haze events. In December 2015, for the first time, the government declared a “red alert” because of air pollution. Due to the emergency measures associated with the red alert, the government closed schools, forced motorists off the road and shut down factories, causing tremendous economic losses. Therefore, in Beijing, haze not only represents an air pollution issue but also reflects a serious socio-economic problem.

Significant air pollution in Beijing has long been a topic of concern^9^. The city’s pollution is related to the massive energy consumption in Beijing and its surrounding areas, mainly in the forms of coal combustion, vehicle emissions, coking and biomass burning^10^,^11^. The battle against air pollution has spanned many years. In 2001, after winning the right to host the 2008 Olympic Games, Beijing increased the pace of air quality improvements by implementing many control measures that focused on modifying Beijing’s energy mix at a high socio-economic cost. For example, a project to convert heating sources from coal to electricity in central downtown Beijing was launched in 2003. Intensive industry relocation activities started in 2005. The current traffic volume control policy started during the 2008 Olympic Games. In 2010, Beijing initiated car purchase restrictions. Recently, the government has been accelerating the conversion of heating sources from coal to natural gas. Whether these costly efforts have been effective is hotly debated^12^. Due to the recent haze problem, the public believes that the air quality is even worse than it was ten years ago. In November 2015, the United Nations Environment Programme (UNEP) and the Beijing Environmental Protection Bureau (BEPB) released a joint report titled “A Review of Air Pollution Control in Beijing: 1998–2013” (www.unep.org/roap/Portals/96/Documents/Air_Pollution_English.pdf). Based on long-term monitoring data of SO_2_, NO_2_, CO and PM_10_, this review found that the control measures are improving Beijing’s air quality. Citywide monitoring of PM_2.5_ started in Beijing after 2012, but the trend of PM_2.5_ during the study period remains unclear. Overall, more studies are needed to evaluate the cost and benefits of Beijing’s air pollution control.

This study used a unique variable, polycyclic aromatic hydrocarbon (PAH) contamination in topsoil, to interpret the evolution of energy consumption and air quality in Beijing. PAHs are a group of toxic and carcinogenic pollutants ubiquitous in urban environments^13^,^14^,^15^,^16^. In densely populated megacities, air pollution by PAHs could result in elevated human health risks^17^,^18^,^19^. PAHs mainly originate from the incomplete combustion of fossil fuels and other organic substances (e.g., biomass) during activities such as industrial production, residential heating, vehicle emissions, power generation, and incineration^20^,^21^,^22^. Particle-bound or gaseous PAHs released from their sources are subject to atmospheric transport. The majority of atmospheric PAHs often contain fine particles smaller than 2.5 μm^23^,^24^. Soil is a major sink of PAHs, receiving these pollutants from both wet and dry atmospheric deposition. In many large cities around the world, the topsoil has been seriously contaminated by PAHs^13^,^25^,^26^,^27^. Many regional studies have shown that the content of PAHs in topsoil is significantly correlated with the content of PAHs in the ambient air^28^,^29^,^30^. Therefore, a study on the evolution of PAH contamination in topsoil may provide valuable information about regional air quality trends.

This study investigated PAH contamination in the topsoil of Beijing’s downtown and suburban areas based on a unique dataset from two years. Two large field campaigns, one organized in 2004 and the other in 2013, generated two snapshots of the contamination. As most PAH compounds are fairly persistent in natural conditions, the snapshots reflect the average contamination situation prior to the campaigns. By comparing the two snapshots, the evolution of PAH contamination during the past decade was assessed. Source apportionment analyses were performed using Positive Matrix Factorization (PMF) to examine the change of PAH sources from 2004 to 2013. PMF is a receptor model approach that allows the optimal use of available data because it can retain missing data points and/or values that are below the detection limit in its calculation. PMF also considers data uncertainty by assessing the quality and reliability of each data point^31^. The main objectives of this study are the following: 1) to demonstrate whether and how soil contamination reflected the change in Beijing’s energy mix and 2) to achieve independent information about the sources of air pollutants in Beijing. The study results provide supplementary evidence for the resolution of ongoing debates related to Beijing’s air pollution (e.g., whether the pollution has been alleviated) and its origins (e.g., whether it is dominated by coal burning or vehicle emissions); the results also generate insights into current and potential future policy measures.

## Results

### PAH concentrations

**Figure 1:**
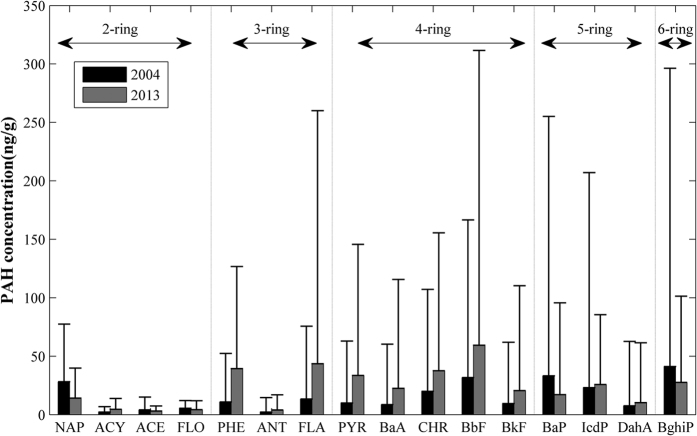
Concentrations of 16 individual PAH compounds in the topsoil. The bars indicate the medians, and the lines above the bars represent the corresponding standard deviations.

Our study focused on the downtown and suburban districts of Beijing. All 16 PAH compounds were detected in the soil samples (45 samples in each campaign) collected within the study area. In the downtown districts, the median concentrations of ∑16 PAHs in 2004 and 2013 were 699.44 ng/g and 500.27 ng/g, respectively. In the suburban districts, the median concentrations were 183.20 ng/g and 177.11 ng/g, respectively, indicating a much lower level of PAH contamination than in the downtown districts. Figure 1 illustrates the concentrations of the 16 compounds in the two periods. As the concentrations have highly skewed distributions, the median concentrations rather than mean values are presented in the figure. To examine whether the concentrations had a statistically significant change from 2004 to 2013, Mann-Whitney U tests were performed, and the results are presented in Table 1. In the table, “All” indicates that both downtown soil samples and suburban soil samples were considered, while “Downtown” and “Suburban” refer to the respective subsets of samples. Although the content of ∑16 PAHs did not change significantly (see Table 1), the 16 PAH compounds showed different trends over time (see Fig. 1 and Table 1). Most of the 3-ring and 4-ring compounds increased over time, whereas most of the 5-ring and 6-ring compounds decreased over time. However, the 2-ring compounds showed no general tendencies. Because 2-ring PAHs are the least persistent compounds, soil samples collected in the field campaigns may not reflect the long-term average conditions of such compounds. The implications of the changes are two-fold: first, the source characteristics of soil PAHs varied, and second, the environmental risk associated with the soil contamination changed. The following subsections further discuss these issues.

### Source apportionment results

**Figure 2:**
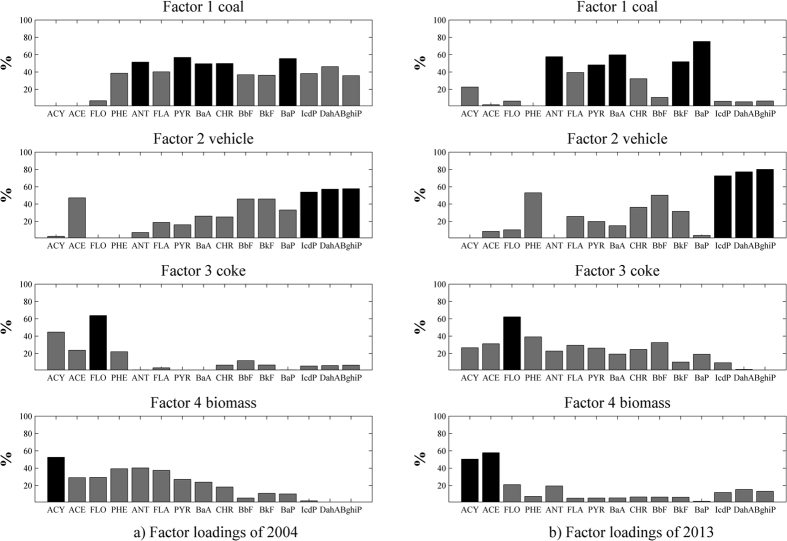
Factor loadings derived in the PMF analysis for the soil samples collected in (**a**) 2004 and (**b**) 2013.

The source profiles derived for both 2004 and 2013 are given in Fig. 2. Each subplot in the figures illustrates the loading of a source factor on different PAH compounds. The PAHs with relatively high loadings are represented by the darker bars. The interpretation of the source factors was based on the prior knowledge that coal combustion, vehicle emissions, coking and biomass burning are the major origins of PAHs in Beijing. In 2004 (Fig. 2a), Factor 1 has high loadings on ANT, PYR, BaA, CHR and BaP. These compounds, as well as FLA, BbF and BkF, are markers for coal combustion^32^,^33^,^34^,^35^,^36^. Therefore, Factor 1 in Fig. 2a can be interpreted as the coal combustion source. Factor 2 is dominated by IcdP, DahA and BghiP. Both BghiP and IcdP have been identified as typical tracers of a vehicular source of PAHs^35^,^36^, and DahA is also associated with traffic emissions^37^. Factor 2 in Fig. 2a can therefore be attributed to vehicle emissions. Factor 3 is mostly associated with FLO, which is a typical marker for coke oven sources^34^,^36^. Therefore, Factor 3 in Fig. 2a represents coking sources. Factor 4 has high loadings of ACY, which is the dominant PAH compound released during biomass burning^38^,^39^. Factor 4 in Fig. 2a can therefore be interpreted as the biomass burning source. Similarly, Factors 1, 2, 3 and 4 in Fig. 2b can be interpreted as coal combustion, vehicle emissions, coking and biomass burning, respectively.

**Figure 3:**
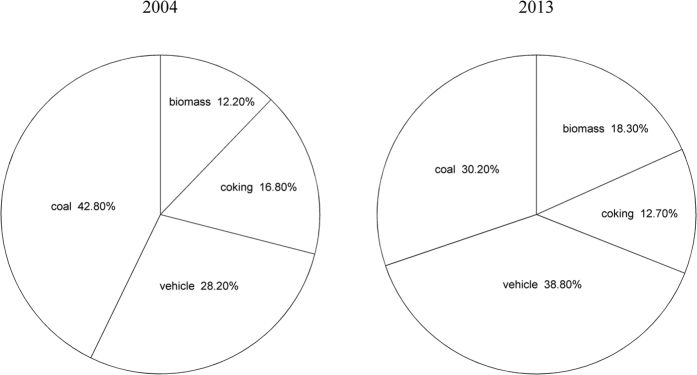
Estimated contributions of different sources in 2004 and 2013.

Figure 3 shows the contributions of the different sources as quantified by the PMF analyses. It is clear that the source composition experienced a significant change from 2004 to 2013. In 2004, the leading PAH source was coal combustion (42.8%), followed by vehicle emissions (28.2%), coking (16.8%) and biomass burning (12.2%). However, in 2013, the contributions of vehicle emissions (38.8%) and biomass burning (18.3%) increased, while those of coal combustion (30.2%) and coking (12.7%) decreased. The order of the four sources changed to vehicle emissions > coal combustion > biomass burning > coking.

### Spatial distributions of risk level

**Figure 4:**
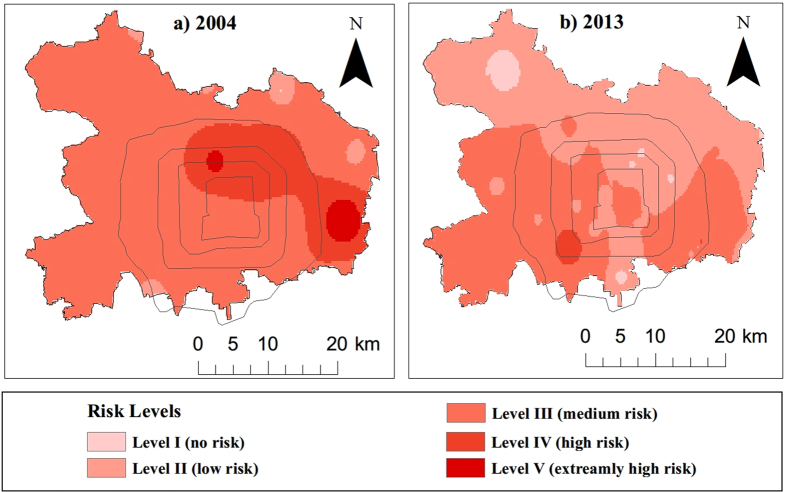
Derived risk levels of soil contamination by PAHs in the downtown area of Beijing based on the carcinogenic equivalent concentration of BaP (i.e., BaP_eq_). The four circles indicate the main ring roads in downtown Beijing (i.e., 2-ring, 3-ring, 4-ring and 5-ring roads, from inside to outside). (**a**) The risk level distribution in 2004; and (**b**) the risk level distribution in 2013. The maps were created using ArcGIS for Desktop (version 10.2.1, http://www.esri.com/software/arcgis/arcgis-for-desktop).

We used the carcinogenic equivalent concentration of BaP (denoted as BaP_eq_) as a risk indicator. The BaP_eq_ concentration mainly reflects the magnitude of coal combustion and vehicle emission sources. The average BaP_eq_ values of all samples were 139.48 ng/g and 310.42 ng/g for 2013 and 2004, respectively, indicating a significant reduction of the potential risk associated with soil contamination. Figure 4 illustrates the spatial distributions of risk level in downtown Beijing based on spatially interpolated BaP_eq_ values. The interpolation was finished in ArcGIS for Desktop (version 10.2.1) using the inverse distance weighting method. As shown in the figure, the risk level substantially decreased from 2004 to 2013 for most of the downtown area, and its spatial pattern changed significantly. In 2004, two contamination hotspots were in the southeastern and northern regions (Fig. 4a). The south-eastern region was once an industrial center, and the northern region is a major residential area. Intensive coal consumption occurred in both regions during the early 2000s. The two hotspots disappeared in 2013 (Fig. 4b), which should be attributed to Beijing’s pollution prevention efforts, as explained below. Many high-pollution plants in the southeastern region, such as Beijing Coking and Chemical Works, Beijing Chemical Works, and Corrosion-proof Railway Sleeper Plant, were relocated outside of the downtown area before the 2008 Olympic Games. On the other hand, during the past decade, the primary energy sources for residential heating in downtown Beijing have shifted away from being dominantly coal to what now being mainly natural gas and electricity. The new hotspot in the southwestern region may be related to increased vehicle emissions in this region. A possible cause of this increase could be the establishment of a second-hand vehicle market in 2002. This market has developed into the largest market of used vehicles in Beijing. The traffic volume in this region has substantially increased due to the expansion of this market, which may explain the new hotspot in 2013.

## Discussion

**Figure 5:**
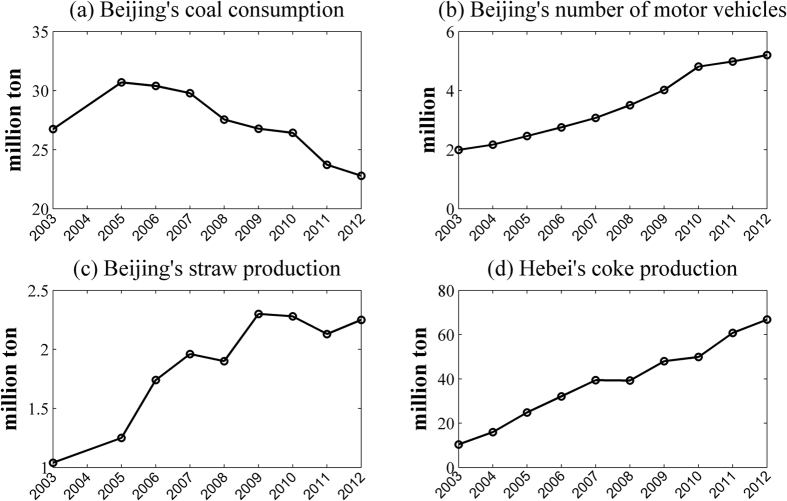
Annual statistics (2003–2012) of (**a**) Beijing’s coal consumption; (**b**) Being’s number of motor vehicles; (**c**) Beijing’s straw production; and (**d**) Hebei’s coke production. The 2004 statistics are missing for Beijing’s coal consumption and straw production.

The PAH contamination in topsoil can be viewed as an indicator of local and regional energy consumption. According to the Beijing Statistical Yearbooks (2004–2013), the total annual energy consumption of Beijing increased from 46.482 million tons of SCE (Standard Coal Equivalent) in 2003 to 71.777 million tons of SCE in 2012. The energy mix also changed significantly. The annual coal consumption in Beijing has been decreasing since 2005 (Fig. 5a), and the number of motor vehicles in 2012 was almost three times the number present in 2003 (Fig. 5b). These observations are consistent with the key result illustrated by Fig. 3: the leading source of soil PAHs shifted from coal combustion to vehicle emissions from 2004 to 2013. In China, straw disposal is still mainly conducted by in-field burning, and straw burning (a major type of biomass burning in China) has been increasingly criticized as an important contributor to haze events in China. Figure 5c shows that the straw production in Beijing doubled from 2003 to 2012^40^,^41^, which coincides with the increased contribution of biomass burning (Fig. 3). Coke production in Beijing decreased from approximately 3.80 million tons per year in 2003 to zero in 2008 (Beijing Statistical Yearbooks, 2004–2013), which explains the decrease of coking’s contribution, as shown in Fig. 3. Although coke production in Beijing was eliminated, the contribution of coking to soil PAHs was still significant (12.7%) as of 2013. Studies^42^,^43^ have shown that the half-life of FLO (the key marker of coking sources) is typically shorter than 100 days. In other words, less than 0.01% of the FLO added to soil would remain in the soil after four years of natural degradation. As of 2013, the coking factories in Beijing had been shut down for more than four years; therefore, the FLO content in Beijing’s topsoil should mainly reflect recent sources instead of historical ones. Thus, the contribution of coking in 2013 is mainly from the coke production in Beijing’s surrounding areas, specifically Heibei Province. Based on the Hebei Economic Yearbooks (2004–2013), the coke production of Heibei Province increased from 10.37 million tons in 2003 to 66.78 million tons in 2012 (Fig. 5d).

**Figure 6:**
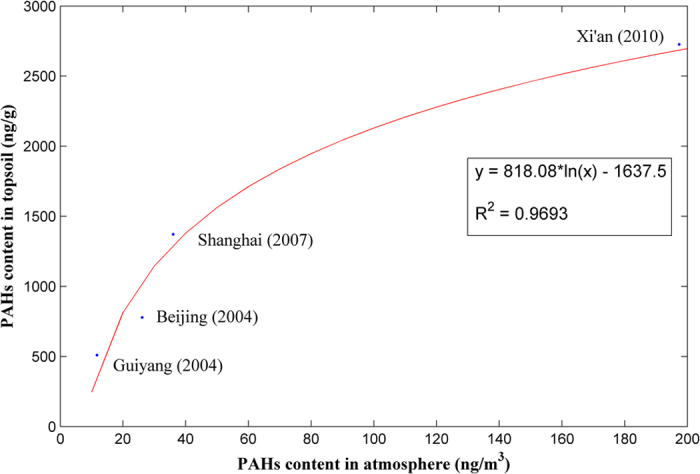
Correlation between PAH contents in topsoil and atmosphere based on data from four of China’s large cities: Guiyang (data year: 2004), Beijing (data year: 2004), Shanghai (data year: 2007), and Xi’an (data year: 2010). The Guiyang data point represents the average content of 365 atmospheric samples and 13 topsoil samples, considering all 16 USEPA priority PAHs except ACY and IcdP. The Beijing data point represents the average content of 44 atmospheric samples and 161 topsoil samples, considering all 16 PAHs. The Shanghai data point represents the average content of 78 atmospheric samples and 54 topsoil samples, considering all 16 PAHs except NAP, ACE and ACY. The Xi’an data point represents the average content of 88 atmospheric samples and 5 topsoil samples, considering all 16 PAHs.

Many studies have been conducted on the atmospheric PAHs in Beijing from 2004 to 2013. Source apportionment has also been attempted. A study^11^ found that fossil fuel uses (mainly coal combustion and vehicle emissions) contributed approximately 65% of the atmospheric PAHs in 2004. A study in 2008^44^ estimated that coal combustion and vehicle emissions accounted for 69% of atmospheric PAHs. A study in 2011^45^ reported that the combined contribution of coal combustion and vehicle emissions was 83% during non-heating seasons. Furthermore, as discussed in the introduction, atmospheric PAHs are mainly distributed in fine particles smaller than 2.5 μm. The UNEP and BEPB’s joint report, as mentioned in the introduction, estimated that motor vehicle and coal consumption contribute 31.1% and 22.4% of PM_2.5_ in Beijing, respectively. All the above results are consistent with our findings on soil PAHs (Fig. 3). In addition, we analyzed the correlation between topsoil and atmospheric PAH content in China’s large cities based on data from the literature^46^,^47^,^48^,^49^,^50^,^51^. Figure 6 demonstrates a strong correlation and a clear logarithmic relationship between PAH content in the soil and air. However, because the sample size is small, the regression function presented in Fig. 6 may not be considered a general relationship; more data are needed to derive a function that is widely applicable. The above evidence reinforces the theory that the PAH contamination in topsoil is closely related to air pollution and could provide valuable, independent information about air pollution.

In summary, this study, based on soil sampling campaigns in 2004 and 2013, investigated the PAH contamination in Beijing topsoil over the past decade. The study results show that although the total PAH content in the topsoil did not significantly decrease from 2004 to 2013, the PAH composition changed considerably, decreasing environmental risk. The source apportionment analyses revealed a notable decrease in the contribution of coal combustion to PAH contamination as well as a significant increase in the contribution of vehicle emissions. Vehicle emissions have become the leading cause of PAH contamination in soil. The source apportionment results clearly reflect the change in Beijing’s energy mix and are consistent with previous studies on Beijing’s atmospheric PAHs. Therefore, we argue that the evolution of PAH contamination in topsoil is indicative of the long-term variation of Beijing’s air quality.

The study results have many important policy implications. First, from the perspective of soil contamination, Beijing’s tremendous efforts to control pollution over the past decade, mainly aimed at shifting its energy mix, had a positive effect. Second, although our investigation into soil contamination did not directly address the issue of PM_2.5_, it offered a unique perspective that could be used to determine whether vehicle emissions or coal is a more important source of Beijing’s PM_2.5_. Our results support the viewpoint that vehicle emissions are currently the most important combustion source. We further argue that the control measures on vehicle purchasing and traffic volume in Beijing, which have been highly controversial, are relevant although not necessarily cost effective. Third, straw burning is a very traditional waste disposal practice in rural China and is a major form of biomass burning in Beijing and its vicinities. Whether straw burning significantly contributes to Beijing’s haze events has been of great public concern. Our study offers indirect evidence of the increasing effects of straw burning (refer to Fig. 3). Appropriate policy measures are therefore desired to control these burning practices. Fourth, although coking has been eliminated in Beijing, its influence on PAH contamination is still considerable due to the rapid growth of coke production in Beijing’s surrounding areas. An important implication of this finding is that local efforts may not be sufficient to tackle Beijing’s pollution problems, and regional collaboration is important.

Overall, our study demonstrated that PAH contamination in topsoil serves as a key indicator of a megacity’s energy consumption and overall environmental quality and should therefore receive continued investigation.

## Data and Methods

### Study area and sampling locations

**Figure 7:**
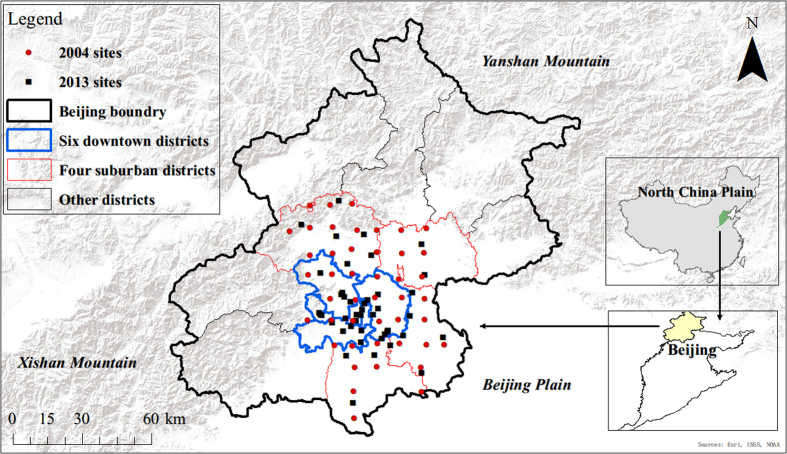
The study area and sampling locations. The map was created using ArcGIS for Desktop (version 10.2.1, http://www.esri.com/software/arcgis/arcgis-for-desktop). The background terrain image was retrieved from ArcGIS Online (http://www.esri.com/software/arcgis/arcgisonline).

Beijing is situated in the north of the North China Plain (Fig. 7) with a sub-humid continental monsoon climate. Its mean annual temperature is approximately 11.6 °C, and its mean annual precipitation is approximately 546 mm. Among the sixteen administrative districts of Beijing, the central six districts (the blue polygons in Fig. 7) represent the downtown area, and the other ten districts are suburban and rural areas. The total population of Beijing was 20.69 million in 2012, of which 59% was in the downtown districts. As the population, traffic, and industrial activities are mainly distributed in Beijing’s plain area, this study considered the six downtown districts and the four suburban districts (the red polygons in Fig. 7) adjacent to them.

The soil samples were collected in two field campaigns. The later campaign was organized in June 2013. A total of 45 topsoil samples were obtained, 28 of which were within the downtown districts and 17 of which were from the four suburban districts (see Fig. 7). Most of the samples were collected in the plain area, and only two were collected in the low-mountain area near the edge of the plain. The sampling locations cover representative land uses in Beijing, including residential areas (10 samples), transportation areas (i.e., large roads, 7 samples), commercial areas (5 samples), recreational areas (i.e., urban parks, 5 samples), industrial sites (8 samples), agriculture land (8 samples) and forests (2 samples). The earlier campaign was conducted from April to June 2004, and its details were reported in a previous study^47^. The 2004 campaign investigated a much larger area than that of our study. From the 2004 study, we considered only the 45 samples from the areas that were also evaluated in the 2013 study: 20 in the downtown districts and 25 in the suburban districts. Compared with the 2013 campaign, the sampling locations in 2004 were more uniformly distributed spatially, and the major land uses were well represented. Both sample sets adequately covered the entire study domain, and they comprise a unique two-year dataset.

### Collection and processing of soil samples

The soil sample collection methods and processing used in 2013 were consistent with those of the 2004 study^47^. The sampling was conducted on dry days with no significant antecedent rainfall events. In the mostly impervious downtown area, samples were collected from green spaces. Our pre-sampling investigation ensured that all of the sampling plots were not notably disturbed for at least one year. At each sampling plot, plant materials were first removed from the soil, and soil samples of the top 10 cm were taken from multiple spots (four corners plus the center) and mixed. The collected samples were stored in stainless steel containers and transferred back to the laboratory in a timely manner. In the laboratory, the samples were air-dried at room temperature. Any large pieces of debris (e.g., gravel or plant residues) were removed from the dried samples. The samples were then ground sufficiently fine to pass through a 1-mm stainless steel sieve. After pretreatment, the soil was air-dried at room temperature and then mixed together completely. The soil was stored in sealed stainless steel containers for further processing. Ten grams of soil sample mixed with 10 g of baked anhydrous sodium sulfate was extracted using accelerated solvent extraction (Dionex ASE 300). Extractions were performed using 34 mL of solvent (1:1 mixture of hexane and dichloromethane). The extracts were concentrated, solvent-exchanged to hexane, and passed through a silica column chromatography. The column was eluted with 25 mL hexane and then 50 mL of dichloromethane:hexane (3:2) mixture. The combined collected solvent was evaporated using a rotary evaporator. The final extracts were concentrated to 1 mL, and known quantities of internal standards 2-fluorobiphenyl and *p*-terphenyl-d_14_ were added and then transferred into vials before analysis.

### Laboratory analysis and quality control

All the samples were analyzed for the following 16 USEPA priority PAHs (two-ring to six-ring compounds): naphthalene (NAP), acenaphthene (ACE), acenaphthylene (ACY), fluorene (FLO), phenanthrene (PHE), anthracene (ANT), fluoranthene (FLA), pyrene (PYR), benz(a)anthracene (BaA), chrysene (CHR), benzo(b)fluoranthene (BbF), benzo(k)fluoranthene (BkF), benzo(a)pyrene (BaP), indeno(l,2,3-cd)pyrene (IcdP), dibenz(a,h)anthracene (DahA), and benzo(g,h,i)perylene (BghiP).

The laboratory sample analysis and quality control used in 2004 were described in a previous paper^47^ and, the same methods were used for the samples in 2013. The PAH concentrations in the extracts were determined by an Agilent 6890 gas chromatograph (Agilent, USA) equipped with a 5973N mass selective detector. The identity of each PAH was confirmed using a standard PAH mixture (610/525/550 in methanol from Chem Service, USA) containing the 16 PAHs. The standard PAH mixture was analyzed in the GC/MS at full scan mode. An HP-5 silica fused capillary column (30 m × 250 μm inner diameter × 250 μm film thickness) was used with a carrier gas of helium at a constant flow rate of 1 mL min^−1^. The oven temperature was programmed to increase from 60 °C to 280 °C at a rate of 5 °C min^−1^ and maintained at 280 °C for 20 min. All solvents, hexane and dichloromethane, were HPLC- grade (CNW Technologies GmbH, Germany). Silica gel (60–80 mesh, Beijing Chemical Reagent Co., China) was heated at 450 °C for 4 hours, kept in a sealed desiccator, and reactivated at 130 °C for 16 hours prior to use. Sodium sulfate was pre-baked at 650 °C for 4 hours and then kept in a sealed desiccator. The working standard solution was prepared by diluting a stock standard from a commercial mixed standard (J&K Chemical Ltd., USA) with hexane.

All data were subject to strict quality control procedures. Quantification was performed using an internal calibration method. The samples were spiked with five surrogate standards (naphthalene-d_8_, acenaphthene-d_10_, phenanthrene-d_10_, chrysene-d_12_, and perylene-d_12_), and the surrogate recoveries were 65% to 127%. For each batch of samples, two procedural blanks were processed, and all data were blank-corrected. The relative percent difference between individual PAH compounds identified in method duplicate samples was <15%. The detection limits were 0.30 ng/g to 0.60 ng/g based on a 10 g soil sample.

### Positive Matrix Factorization

Positive matrix factorization (PMF) is a principal component analysis (PCA)-based receptor model with non-negative constraints:


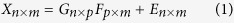


where *X*_*n*×*m*_ is a matrix of sample concentrations that can be decomposed into *G*_*n*×*p*_, a matrix of source contributions, and *F*_*p*×*m*_, a matrix of source profiles, plus a residual matrix, *E*_*n*×*m*_. *n* and *m* denote the number of samples and the number of pollutant species, respectively, and *p* is the number of influential sources. The physical nature of the influential pollution sources can be interpreted from *F*_*p*×*m*_ based on prior information such as measured profiles of specific sources and emission inventories. Each row in *F*_*p*×*m*_ represents an influential source (i.e., a factor), and the sum of the row elements (i.e., factor loadings) quantifies the overall contribution of the source. PMF minimizes the following objective function^52^:


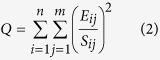


where *S*_*ij*_ represents the “uncertainty” in the *j*th species for the *i*th sample and *E*_*ij*_ (i.e., elements of the residual matrix *E*_*n*×*m*_) represents the error corresponding to S_*ij*_.

In this study, the EPA PMF 5.0 program^53^ was used to conduct PMF analyses. The program calculates *s*_*ij*_ based on the method detection limit (MDL) of targeted pollutant species as:


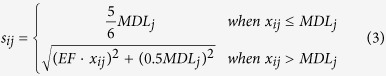


where *x*_*ij*_ represents elements of the concentration matrix *X*_*n*×*m*_ and EF is a measurement error fraction. EF was set to 0.2 in this study. In PMF analyses, the value of *p* has to be carefully determined to ensure physically meaningful results while keeping the matrix dimensionality low^54^. The value of Q, which indicates the agreement of model fit, can help determine an adequate *p* value. The theoretical Q value should be approximately equal to the number of degrees of freedom^55^, as in Eq. (4):


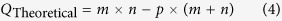


Because NAP is a relatively volatile and unstable compound, it was excluded from the source apportionment, which was aimed at determining the long-term characteristics of PAH sources. Two PMF models were established for the sample sets of 2004 and 2013. *p* was varied between 3 and 6 and eventually set to 4, for which the Q values calculated by Eq. (2) (429.9 for 2013 and 438.3 for 2004) best approximate the theoretical Q value (equal to 435) calculated by Eq. (4).

### Risk levels

We used the carcinogenic equivalent concentration of BaP (denoted as BaP_eq_) as a risk indicator^56^. BaP_eq_ can be calculated as follows:


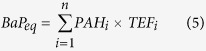


where TEF_*i*_ is the toxic equivalency factor for the *i*th PAH compound (i.e., PAH_*i*_). The TEF values are 0.001 for NAP, ACY, ACE, FLO, PHE, FLA and PYR; 0.01 for ANT, CHR and BghiP; 0.1 for BaA, BbF, BkF and IcdP; and 1 for BaP and DahA^57^. In China, the existing Environmental Quality Standards for Soils (GB15618-1995) does not have content limits for soil PAHs. A new version (GB15618-2008) of this national standard is currently under discussion but has not been officially released yet. This unreleased standard defines four content limits for BaP in soils: I) BaP ≤ 10 ng/g (background condition); II) 10 ng/g < BaP ≤ 100 ng/g (good for agricultural uses); III) 100 ng/g < BaP ≤ 500 ng/g (good for residential land uses); and IV) 500 ng/g < BaP ≤ 1000 ng/g (good for industrial and commercial uses). In this study, we compared the derived BaP_eq_ values to these limits and defined five levels of risk: I) BaP_eq_ ≤ 10 ng/g (no risk); II) 10 ng/g < BaP_eq_ ≤ 100 ng/g (low risk); III) 100 ng/g < BaP_eq_ ≤ 500 ng/g (medium risk); IV) 500 ng/g < BaP_eq_ ≤ 1000 ng/g (high risk); and V) BaP_eq_ > 1000 ng/g (extremely high risk).

## Additional Information

**How to cite this article**: Li, J. *et al.* PAH contamination in Beijing’s topsoil: A unique indicator of the megacity’s evolving energy consumption and overall environmental quality. *Sci. Rep.* **6**, 33245; doi: 10.1038/srep33245 (2016).

## Figures and Tables

**Table 1:** Results of the Mann-Whitney U tests.

PAH	Ring number	All	Downtown	Suburban
NAP	2	↓↓	↓↓	↓↓
ACY	2	↑↑	—	↑↑
ACE	2	—	↓↓	—
FLO	2	—	—	—
PHE	3	↑↑	↑	—
ANT	3	↑↑	—	↑↑
FLA	3	↑↑	—	↑
PYR	4	↑↑	—	↑
BaA	4	↑	—	—
CHR	4	—	—	—
BbF	4	—	—	—
BkF	4	↑	—	—
BaP	5	↓↓	↓↓	↓↓
IcdP	5	—	↓	—
DahA	5	—	—	—
BghiP	6	↓	↓↓	↓↓
∑16 PAHs	2 to 6	—	—	—

“↓↓” and “↑↑” indicate highly significant (*p*-value equals to 0.01) decreases and increases between 2004 and 2013, respectively; “↓” and “↑” indicate significant (*p*-value equals to 0.05) decreases and increases, respectively; and “—” indicates no significant changes.

## References

[b1] Cyranoski D. Satellite view alerts China to soaring pollution. Nature 437, 12 (2005). 16136098 10.1038/437012b

[b2] Calderón-Garcidueñas L., Kulesza R. J., Doty R. L., D’Angiulli A. & Torres-Jardón R. Megacities air pollution problems: Mexico City Metropolitan Area critical issues on the central nervous system pediatric impact. Environmental Research 137, 157–169 (2015). 25543546 10.1016/j.envres.2014.12.012

[b3] Lelieveld J., Evans J. S., Fnais M., Giannadaki D. & Pozzer A. The contribution of outdoor air pollution sources to premature mortality on a global scale. Nature 525, 367–371 (2015). 26381985 10.1038/nature15371

[b4] Seinfeld J. H. Air pollution: A half century of progress. Aiche Journal 50, 1096–1108 (2004).

[b5] Huang R.-J. *et al.* High secondary aerosol contribution to particulate pollution during haze events in China. Nature 514, 218–222 (2014). 25231863 10.1038/nature13774

[b6] Li P. *et al.* Reinstate regional transport of PM2.5 as a major cause of severe haze in Beijing. Proceedings of the National Academy of Sciences 112, E2739–E2740 (2015). 10.1073/pnas.1502596112 PMC4450418 25941410

[b7] Gao J. *et al.* The variation of chemical characteristics of PM2.5 and PM10 and formation causes during two haze pollution events in urban Beijing, China. Atmospheric Environment 107, 1–8 (2015).

[b8] Yang Y. *et al.* Formation mechanism of continuous extreme haze episodes in the megacity Beijing, China, in January 2013. Atmospheric Research 155, 192–203 (2015).

[b9] Swinbanks D. Beijing media join attack on air pollution. Nature 392, 853–853 (1998).9582061

[b10] Song Y. *et al.* Source apportionment of PM2.5 in Beijing in 2004. Journal of hazardous materials 146, 124–130 (2007). 17208371 10.1016/j.jhazmat.2006.11.058

[b11] Zhou J. *et al.* Composition and sources of organic matter in atmospheric PM 10 over a two year period in Beijing, China. Atmospheric Research 93, 849–861 (2009).

[b12] Chen Y., Jin G. Z., Kumar N. & Shi G. The promise of Beijing: Evaluating the impact of the 2008 Olympic Games on air quality. Journal of Environmental Economics and Management 66, 424–443 (2013).

[b13] Agarwal T., Khillare P. S., Shridhar V. & Ray S. Pattern, sources and toxic potential of PAHs in the agricultural soils of Delhi, India. Journal of hazardous materials 163, 1033–1039 (2009). 18757133 10.1016/j.jhazmat.2008.07.058

[b14] Wang J. *et al.* Long term observations of PM2.5-associated PAHs: Comparisons between normal and episode days. Atmospheric Environment 104, 228–236 (2015).

[b15] Mielke H. W. *et al.* PAHs and metals in the soils of inner-city and suburban New Orleans, Louisiana, USA. Environmental Toxicology and Pharmacology 18, 243–247 (2004). 21782755 10.1016/j.etap.2003.11.011

[b16] Nam J. J. *et al.* PAHs in background soils from Western Europe: Influence of atmospheric deposition and soil organic matter. Chemosphere 70, 1596–1602 (2008). 17888489 10.1016/j.chemosphere.2007.08.010

[b17] Zhang Y., Tao S., Shen H. & Ma J. Inhalation exposure to ambient polycyclic aromatic hydrocarbons and lung cancer risk of Chinese population. Proceedings of the National Academy of Sciences 106, 21063–21067 (2009). 10.1073/pnas.0905756106 PMC2789752 19995969

[b18] Bostrom C. E. *et al.* Cancer risk assessment, indicators, and guidelines for polycyclic aromatic hydrocarbons in the ambient air. Environmental health perspectives 110 Suppl 3, 451–488 (2002). 12060843 10.1289/ehp.110-1241197 PMC1241197

[b19] Cheng J. *et al.* PM10-bound Polycyclic Aromatic Hydrocarbons (PAHs) and Cancer Risk Estimation in the Atmosphere Surrounding an Industrial Area of Shanghai, China. Water Air Soil Pollut 183, 437–446 (2007).

[b20] Garban B., Blanchoud H., Motelay-Massei A., Chevreuil M. & Ollivon D. Atmospheric bulk deposition of PAHs onto France: trends from urban to remote sites. Atmospheric Environment 36, 5395–5403 (2002).

[b21] Dyke P. H., Foan C. & Fiedler H. PCB and PAH releases from power stations and waste incineration processes in the UK. Chemosphere 50, 469–480 (2003). 12685746 10.1016/s0045-6535(02)00627-6

[b22] Mastral A. M. *et al.* Spatial and temporal PAH concentrations in Zaragoza, Spain. Science of the Total Environment 307, 111–124 (2003). 12711429 10.1016/S0048-9697(02)00460-6

[b23] Gupta S., Kumar K., Srivastava A., Srivastava A. & Jain V. K. Size distribution and source apportionment of polycyclic aromatic hydrocarbons (PAHs) in aerosol particle samples from the atmospheric environment of Delhi, India. Science of The Total Environment 409, 4674–4680 (2011). 21889785 10.1016/j.scitotenv.2011.08.008

[b24] Wu S. P., Tao S. & Liu W. X. Particle size distributions of polycyclic aromatic hydrocarbons in rural and urban atmosphere of Tianjin, China. Chemosphere 62, 357–367 (2006). 15982711 10.1016/j.chemosphere.2005.04.101

[b25] Jiang Y.-F. *et al.* Levels, composition profiles and sources of polycyclic aromatic hydrocarbons in urban soil of Shanghai, China. Chemosphere 75, 1112–1118 (2009). 19201443 10.1016/j.chemosphere.2009.01.027

[b26] Zhang H. B., Luo Y. M., Wong M. H., Zhao Q. G. & Zhang G. L. Distributions and Concentrations of PAHs in Hong Kong Soils. Environmental pollution 141, 107–114 (2006). 16242223 10.1016/j.envpol.2005.08.031

[b27] Kwon H.-O. & Choi S.-D. Polycyclic aromatic hydrocarbons (PAHs) in soils from a multi-industrial city, South Korea. Science of The Total Environment 470–471, 1494–1501 (2014). 10.1016/j.scitotenv.2013.08.031 24011990

[b28] Vogt N. B. *et al.* Polycyclic aromatic hydrocarbons in soil and air: statistical analysis and classification by the SIMCA method. Environmental science & technology 21, 35–44 (1987).

[b29] Amagai T. *et al.* A survey on polycyclic aromatic hydrocarbon concentrations in soil in Chiang-Mai, Thailand. Environment international 25, 563–572 (1999).

[b30] Trapido M. Polycyclic aromatic hydrocarbons in Estonian soil: contamination and profiles. Environmental pollution 105, 67–74 (1999).

[b31] Yang B. *et al.* Source apportionment of polycyclic aromatic hydrocarbons in soils of Huanghuai Plain, China: Comparison of three receptor models. Science of The Total Environment 443, 31–39 (2013). 23178888 10.1016/j.scitotenv.2012.10.094

[b32] Duval M. & Friedlander S. Source resolution of polycyclic aromatic hydrocarbons in the Los Angeles atmosphere application of a CMB with first-order decay. *US EPA Report EPA-600*/*2*-*81*-*161*, *US Government Printing Office, Washington, DC, USA* (1981).

[b33] Harrison R. M., Smith D. & Luhana L. Source apportionment of atmospheric polycyclic aromatic hydrocarbons collected from an urban location in Birmingham, UK. Environmental Science & Technology 30, 825–832 (1996).

[b34] Simcik M. F., Eisenreich S. J. & Lioy P. J. Source apportionment and source/sink relationships of PAHs in the coastal atmosphere of Chicago and Lake Michigan. Atmospheric Environment 33, 5071–5079 (1999).

[b35] Larsen R. K. & Baker J. E. Source apportionment of polycyclic aromatic hydrocarbons in the urban atmosphere: a comparison of three methods. Environmental Science & Technology 37, 1873–1881 (2003). 12775060 10.1021/es0206184

[b36] Zuo Q., Duan Y., Yang Y., Wang X. & Tao S. Source apportionment of polycyclic aromatic hydrocarbons in surface soil in Tianjin, China. Environmental pollution 147, 303–310 (2007). 16828945 10.1016/j.envpol.2006.05.029

[b37] Fraser M. P., Cass G. R., Simoneit B. R. & Rasmussen R. Air quality model evaluation data for organics. 4. C2-C36 non-aromatic hydrocarbons. Environmental science & technology 31, 2356–2367 (1997). 10.1021/es0209262 12630457

[b38] Khalili N. R., Scheff P. A. & Holsen T. M. PAH source fingerprints for coke ovens, diesel and, gasoline engines, highway tunnels, and wood combustion emissions. Atmospheric environment 29, 533–542 (1995).

[b39] Shao Y. *et al.* Occurrence and source apportionment of PAHs in highly vulnerable karst system. Science of The Total Environment 490, 153–160 (2014). 24852613 10.1016/j.scitotenv.2014.04.128

[b40] Cao G., Zhang X., ZHENG F. & Wang Y. Estimating the Quantity of Crop Residues Burnt in Open Field in China. Resources Science 28, 9–13 (2006).

[b41] Wang M., Liu S. & Shi M. The estimation and prediction of PM2.5 emission in Beijing: a case of straw burning. Practical Electronics 9, 245-245, 230 (2013).

[b42] Banach-Szott M., Debska B., Wisniewska A. & Pakula J. Changes in the contents of selected polycyclic aromatic hydrocarbons in soils of various types. Environmental Science and Pollution Research International 22, 5059–5069 (2015). 25586610 10.1007/s11356-014-3901-9 PMC4366570

[b43] Oleszczuk P. & Baran S. Degradation of individual polycyclic aromatic hydrocarbons (PAHs) in soil polluted with aircraft fuel. Polish Journal of Environmental Studies 12, 431–438 (2003).

[b44] Wang X., Cheng H., Xu X., Zhuang G. & Zhao C. A wintertime study of polycyclic aromatic hydrocarbons in PM 2.5 and PM 2.5–10 in Beijing: assessment of energy structure conversion. Journal of hazardous materials 157, 47–56 (2008). 18342441 10.1016/j.jhazmat.2007.12.092

[b45] Ma W.-L. *et al.* Atmospheric concentrations, sources and gas-particle partitioning of PAHs in Beijing after the 29th Olympic Games. Environmental pollution 159, 1794–1801 (2011). 21497969 10.1016/j.envpol.2011.03.025

[b46] Duan F., He K. & Ma Y. Concentration and sources of atmospheric polycyclic aromatic hydrocarbons (PAHs) in PM2.5 in Beijing. Acta Scientiae Circumstantiae 29, 1363–1371 (2009).

[b47] Wang K. *et al.* Application of spatial analysis and multivariate analysis techniques in distribution and source study of polycyclic aromatic hydrocarbons in the topsoil of Beijing, China. Environmental geology 56, 1041–1050 (2009).

[b48] Wang X. Y. *et al.* Characteristics and sources of atmospheric polycyclic aromatic hydrocarbons (PAHs) in Shanghai, China. Environ Monit Assess 165, 295–305 (2010). 19440848 10.1007/s10661-009-0946-1

[b49] Liu Y. *et al.* Polycyclic aromatic hydrocarbons in the surface soil of Shanghai, China: concentrations, distribution and sources. Organic Geochemistry 41, 355–362 (2010).

[b50] Hu J., Zhang G. & Lui C.-Q. Pilot study of polycyclic aromatic hydrocarbons in surface soils of Guiyang city, People’s Republic of China. Bulletin of environmental contamination and toxicology 76, 80–89 (2006). 16404664 10.1007/s00128-005-0892-8

[b51] Zhang C., Chen J., Liu l., Li W. & Ma W. Seasonal variation and health risk assessment of polycyclic aromatic hydrocarbons in air of Xi’an. Chinese Journal of Environmental Engineering 6, 4579–4584 (2012).

[b52] Paatero P. Least squares formulation of robust non-negative factor analysis. Chemometrics and intelligent laboratory systems 37, 23–35 (1997).

[b53] Norris G., Duvall R., Brown S. & Bai S. EPA Positive Matrix Factorization (PMF) 5.0 Fundamentals and User Guide. (US Environmental Protection Agency, Office of Research and Development, Washington, DC, 2014).

[b54] Wang D., Tian F., Yang M., Liu C. & Li Y.-F. Application of positive matrix factorization to identify potential sources of PAHs in soil of Dalian, China. Environmental pollution 157, 1559–1564 (2009). 19201072 10.1016/j.envpol.2009.01.003

[b55] Bzdusek P. A., Christensen E. R., Lee C. M., Pakdeesusuk U. & Freedman D. L. PCB Congeners and Dechlorination in Sediments of Lake Hartwell, South Carolina, Determined from Cores Collected in 1987 and 1998. Environmental Science & Technology 40, 109–119 (2006). 16433340 10.1021/es050194o

[b56] Zheng Y. *et al.* Assessing the polycyclic aromatic hydrocarbon (PAH) pollution of urban stormwater runoff: A dynamic modeling approach. Science of The Total Environment 481, 554–563 (2014). 24631618 10.1016/j.scitotenv.2014.02.097

[b57] Nisbet I. C. & LaGoy P. K. Toxic equivalency factors (TEFs) for polycyclic aromatic hydrocarbons (PAHs). Regulatory toxicology and pharmacology 16, 290–300 (1992). 1293646 10.1016/0273-2300(92)90009-x

